# Performance of community health workers: situating their intermediary position within complex adaptive health systems

**DOI:** 10.1186/s12960-017-0234-z

**Published:** 2017-09-02

**Authors:** Maryse C. Kok, Jacqueline E. W. Broerse, Sally Theobald, Hermen Ormel, Marjolein Dieleman, Miriam Taegtmeyer

**Affiliations:** 1 KIT | Royal Tropical Institute, P.O. Box 95001, 1090 HA Amsterdam, The Netherlands; 2 Athena Institute for Research on Innovation and Communication in Health and Life Sciences, VU University Amsterdam, De Boelelaan 1081, 1081 HV Amsterdam, The Netherlands; 3 Department of International Public Health, Liverpool School of Tropical Medicine, Pembroke Place, Liverpool, L3 5QA United Kingdom

**Keywords:** Community health workers, Performance, Low- and middle-income countries, Complex adaptive systems

## Abstract

Health systems are social institutions, in which health worker performance is shaped by transactional processes between different actors.

This analytical assessment unravels the complex web of factors that influence the performance of community health workers (CHWs) in low- and middle-income countries. It examines their unique intermediary position between the communities they serve and actors in the health sector, and the complexity of the health systems in which they operate. The assessment combines evidence from the international literature on CHW programmes with research outcomes from the 5-year REACHOUT consortium, undertaking implementation research to improve CHW performance in six contexts (two in Asia and four in Africa). A conceptual framework on CHW performance, which explicitly conceptualizes the interface role of CHWs, is presented. Various categories of factors influencing CHW performance are distinguished in the framework: the context, the health system and intervention hardware and the health system and intervention software. Hardware elements of CHW interventions comprise the supervision systems, training, accountability and communication structures, incentives, supplies and logistics. Software elements relate to the ideas, interests, relationships, power, values and norms of the health system actors. They influence CHWs’ feelings of connectedness, familiarity, self-fulfilment and serving the same goals and CHWs’ perceptions of support received, respect, competence, honesty, fairness and recognition.

The framework shines a spotlight on the need for programmes to pay more attention to ideas, interests, relationships, power, values and norms of CHWs, communities, health professionals and other actors in the health system, if CHW performance is to improve.

## Background

Community health workers (CHWs) have a unique intermediary position between communities and the health sector. They form an essential group of health workers in many low- and middle-income countries (LMICs), delivering promotive, preventive and (limited) curative health services. CHWs have been shown to contribute to the improved health of rural and poor communities [1]. There are many types of CHWs, depending on the country and setting. All have in common that they are health workers performing tasks related to healthcare delivery, that they have received some training focused on the activities they need to carry out in the context of the intervention(s) they implement and that they have no formal professional or paraprofessional certificate or tertiary education degree [1]. In some countries, CHWs are salaried workers and an official part of the health sector, while in other countries, they are volunteers at the village level. In many cases, they are drawn from and selected by the communities that they serve. They can have specified tasks and work for a particular programme (for example a malaria or tuberculosis programme), but many CHWs have broad job descriptions with different tasks and high workloads. CHWs often constitute the first point of contact with the health sector for people at the community level [2]. In addition, in many contexts, programmatic expectations include CHWs acting as cultural brokers or mediators, empowering communities to claim (health-related and other) rights [3, 4].

There has been a renewed interest in CHW programmes in recent years, as a result of the continuous shortage of human resources for health and an increasing burden of disease [5, 6]. The shifting of tasks from more to less specialized health workers is taking place in many LMICs, and tasks are often extended to CHWs [7]. However, CHW programmes are often hampered by poor motivation, high workloads and varying quality when brought to scale, resulting in sub-optimal effectiveness [5, 8]. There is a need to better understand the context and conditions in which CHWs work, in order to support them in improving their performance and realizing their potential [1, 8]. This analytical assessment presents a conceptual framework of CHW performance which takes existing models [9–11] into account but argues that there is a need to look at CHW performance from a broader systems and social perspective, given CHWs’ intermediary position between communities and the health sector. Our argument is based on the growing body of evidence on CHW programmes and research outcomes and experiences from the REACHOUT consortium^1^.

## Community health worker performance

Well-performing health workers work in ways that are responsive, fair and efficient to achieve the best health outcomes possible for clients, given available resources and circumstances [12]. Improving the performance of health workers in LMICs is complex, due to the intersection of multiple factors that influence health workers’ ability and willingness to carry out their tasks [13]. The definition of health worker performance and the factors influencing it also apply to CHWs. The complexity of CHW performance lies not only in the multitude of influencing factors but also in the fact that performance—at the individual level—is the sum of different interrelated attributes, such as self-esteem, motivation, attitudes, competencies, guideline adherence, job satisfaction and capacity to facilitate community agency [9]. CHW performance can also be measured in terms of client-related outcomes, such as utilization of health services, and at the same time (some of the attributes of) CHW performance can also be influenced by the behaviours of clients or other actors. This is profound in the case of CHWs, as their relationships with clients and other actors in the community are an explicit focus of their work. Thus, performance is shaped by transactional social processes between CHWs and their environment—both at the community level and in interactions with colleagues within health service delivery [13].

## Factors influencing community health worker performance

Variations in the design of CHW interventions have a significant and direct influence on CHW performance. Our systematic review of the literature, which included 140 studies on CHW programmes, found that multiple intervention design factors, such as different types of supervision, incentives, training, accountability and communication structures, logistics and supplies, influence CHW performance [9]. This and other reviews stress the importance of building elements into the intervention design that facilitate performance from both the health sector and community side [1, 8, 9, 14–16]. We furthermore identified contextual factors that influence CHW performance. These include community context, such as cultural and gender norms, the economy including market forces, the environment, and health system policy and practice (e.g. availability of a CHW and human resources policy and human resources provisions and governance structure) [17]. Both intervention design and contextual factors form an interactive web, influencing performance of CHW programmes through the experiences, mindsets and values that shape the behaviour of actors and their relationships. To further explore the pathways leading to CHW performance, we undertook a qualitative comparative study of the CHW programmes in Ethiopia, Kenya, Malawi and Mozambique. The study included a total of 15 focus group discussions (FGDs) and 38 interviews with CHWs, 70 interviews with CHW supervisors and managers and 33 FGDs and 46 interviews with various types of community members. The intermediary position of CHWs and the concept of performance as a transactional social process led us to focus on how relationships between different actors influenced CHW performance. The study demonstrated a complex interplay of factors influencing trust, and thereby the strength of relationships, between CHWs, their communities and actors in the health sector, such as supervisors and managers. In different contexts, these relationships were shaped through various mechanisms, such as feelings of (dis)connectedness, (un)familiarity, self-fulfilment and serving the same goals and perceptions of support received, respect, competence, honesty, fairness and recognition. CHW performance was clearly related to trusting relationships among different actors, which are often related to experiences regarding power and hierarchy. The socio-economic situation, the history and value of community participation and volunteerism and the role of traditional leaders were found to influence relationships. The programme context, e.g. selection and recruitment systems, extent of task-shifting, volunteering and mode of supervision, was also of influence [18].

## A conceptual framework on community health worker performance

**Fig. 1:**
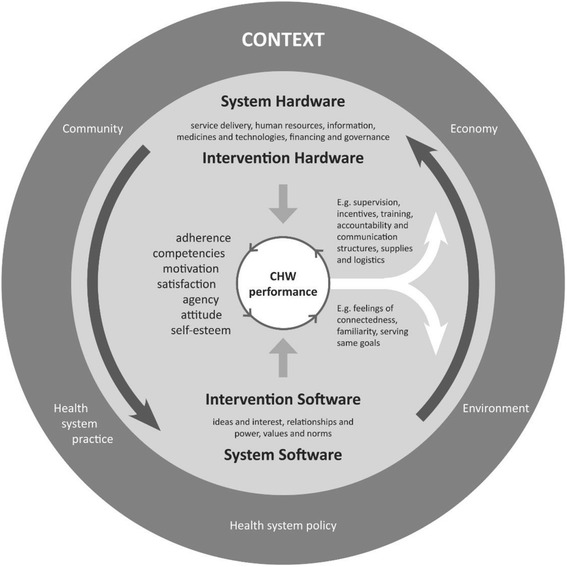
Conceptual framework visualizing CHW performance as a transactional social process. The figure shows the interplay of factors that influence CHW performance: context, system and intervention hard- and software

Perceiving CHW performance as a transactional social process calls for a conceptual framework that recognizes the importance of relationships and power between different actors in the health system (Fig. 1). The framework and its underlying hypotheses focus on CHW performance as a social process, embedded in a health system seen as a social construct—as conceptualized by Sheikh et al. in their article on the development of health policy and systems research [19]. The hypotheses on how the different elements presented in this framework affect each other could be connected to specific pathways leading to improved CHW performance. The framework is based on findings from the international literature on CHW programmes [9, 17] combined with research outcomes from the REACHOUT consortium [18].

The conceptual framework has CHW performance in the centre. Different circles around CHW performance present the multiple layers of influencing factors, starting with the intervention (or programme) design in the inner circle and the health system and broader context in the outer circles. Health system-related influencing factors are divided into “hardware” and “software” [19] and are coupled with hardware and software elements of intervention designs. System hardware includes the six building blocks of the health system framework [20], and they affect intervention design factors, such as the supervision system; training, accountability and communication structures; incentives; supplies; and logistics. The attributes of CHW performance that are mostly influenced by hardware elements are CHWs’ competencies and adherence to guidelines and procedures, in addition to motivation and satisfaction (the attributes are presented at the left side of “CHW performance” for the sake of clarity). System software includes the ideas and interest, relationships and power, values and norms of the actors in the health system and CHW programme [19]. These actors include CHWs, their clients, the community, health professionals and other people in the health sector. The system software influences CHWs’ feelings of connectedness, familiarity, self-fulfilment and serving the same goals and CHWs’ perceptions of support received, respect, competence, honesty, fairness and recognition [18]. These software elements have effects on CHW performance, influencing attributes such as self-esteem, attitudes and agency (which is related to the ability of CHWs to stimulate community agency), in addition to motivation and satisfaction. The latter attributes of CHW performance—motivation and satisfaction—are influenced by both hardware and software elements (and therefore presented in the middle of the list of attributes). The hardware and software elements continuously influence each other (indicated with the big arrows in the circle). For example, the availability and design of the supervision system influences the strength of relationships between CHWs and health sector actors and sometimes also between CHWs and the community [21]. If relationships between CHWs and communities are constrained, intervention design elements (hardware) could be adjusted or introduced to improve software elements. For example, when representatives from existing community networks receive a formal role in identifying challenges in service delivery, testing solutions and monitoring changes within the CHW programme, relationships and thus CHW performance could improve [22].

CHW performance is not static. A different combination of attributes of performance will be present over time, and they do not stand on their own, but influence each other (indicated with the arrows in the small inner circle of CHW performance). The “status” of CHW performance, in other words the constitution of its different attributes, can have a reciprocal effect on the intervention, system and broader context in which CHWs are working (indicated by the two arrows from the centre pointing towards the surrounding circles). Actors in the CHW programme and in the health system and society as a whole have opinions and perceptions about CHW performance, which influence their trust in and relationships with CHWs, again influencing CHW performance. At the hardware side, assessments of guideline adherence or competencies of CHWs can lead to adjustments in how CHWs are trained or supervised, which in turn can influence CHW performance.

It should to be noted that the systematic review [9, 17] and empirical research [18] conducted by REACHOUT did not focus on all hard- and software elements that could influence CHW performance, but many of these elements emerged during the qualitative studies in the six countries. In addition, the attributes of CHW performance were taken from the literature and not individually analysed nor assessed; rather, they were taken as (sometimes self-reported) outcome measures in the respective studies. The conceptual framework does not visualize the effects of CHW performance at the end user and impact level; however, we assume that improved CHW performance leads to improved service delivery, positive changes in health-seeking behaviour and utilization of services by communities and ultimately impacts on the health of the population [9, 10].

## Towards enhanced community health worker performance: interactions between hardware, software and context

Conceptualizing CHW performance as a transactional social process within complex, adaptive health systems has important implications for policy, practice and research. The intermediary position of CHWs between the community and health sector stresses the importance of the (sometimes overlooked) software elements, including trusting relationships between all actors. The realization that hard- and software elements are both needed and can strengthen (or weaken) each other calls for interventions facilitating processes in which the hard- and software elements mutually strengthen each other. It also calls for caution, as the broader context is diverse. For example, CHW programme elements related to hardware can never fully “fix” problems related to the software. Furthermore, both hardware and software can create an enabling environment for performance, but do not always lead to improved performance, as intrinsic factors, such as people’s personalities, play a role.

There are examples of how intervention design can provide for necessary hard- *and* software elements to improve CHW performance. In several settings, the involvement of both the community and health sector in the selection and monitoring of CHWs turns out to improve performance [23]. The establishment of functional structures, such as joint review meetings and village health committees, can facilitate involvement of and trusting relationships between all actors [24]. There is a call for more community voice in CHW programming. Interestingly, CHWs themselves are often believed to be vehicles for facilitating community agency and triggering social change. Some scholars state that this function of CHWs has been pushed from the forefront by technical tasks focusing on attaining disease-specific targets [3, 25, 26]. The task composition, but also the way in which CHW performance is measured (related to hardware), have a bearing on how communities look at CHWs and thereby influence CHWs’ capacity to relate to the community and facilitate agency (related to software). When CHWs are required to act as agents of social change, they themselves need to feel empowered and supported through enabling environments. For example, they need to be trained in soft skills such as communication, problem-solving and assuring confidentiality at community level [27].

Our empirical research shows that many CHWs do not feel supported nor respected by the “upper level”, which hinders motivation and performance. Joint training of CHWs with their supervisors (a hardware element) could contribute to better relationships (part of software), as understanding about each other’s roles and competencies can be established. There is a need for improved, supportive supervision, including training of supervisors in technical skills, people management and implications of CHWs’ intermediate position for relationship building with communities [28, 29]. As supervision is a form of human interaction, strategies that reduce social distance between supervisor and supervisee (such as team building events) could improve relationships and performance. Improved supervision from the side of the health sector could have a positive ripple effect on CHWs’ relationships with their communities, through increased recognition [21].

There is an ongoing debate about whether CHWs should be formally integrated in the health sector [30, 31]. Many LMICs are moving in this direction, often aiming for a mix of paid and voluntary CHWs. Establishing CHWs as a formal cadre in the health sector would require accommodating CHWs’ voices and rights through regulatory frameworks, career paths and worker associations (which can be seen as system hardware). Several studies support remuneration of CHWs when they have multiple tasks that require substantial time investments and are often formerly conducted by health professionals [32]. Remuneration of CHWs with extended tasks can enhance credibility and community trust. However, financial incentives need to be distributed in an equitable and reliable way [33] to avoid mistrust between actors in the health system. Payment of CHWs is an essential motivator, as it contributes to meeting the basic needs for CHWs and their families, who often live in poor areas. However, philosophical considerations and financial realities can be reasons for programmes to continue using volunteers [34]. Some scholars argue that paid CHWs feel more answerable to the organization they are working for than to their communities, which could lead to mistrust and negative effects on CHW performance [34, 35]. In this case, remuneration (a hardware element) could have a negative effect on software elements. Innovative strategies that keep paid CHWs connected to their communities could be developed and studied, and supervision and performance appraisal (with roles for both the health sector and communities) could be organized in such a way that they explicitly stimulate CHWs’ capacity to facilitate community agency.

Programmes that include voluntary, part-time CHWs with limited tasks have shown positive effects [36]. The history and value of volunteerism has been identified as an important contextual factor influencing CHW performance. CHWs can have different motivations to volunteer, such as gaining social respect, religious and moral duty and altruistic concerns for others: software elements which are interlinked with the social and cultural context. However, a recent study showed ambivalence in motivation: uncertainty regarding achieving basic food security and improved socio-economic status for themselves and their families made voluntary CHWs in Ethiopia request for remuneration [37]. Voluntary CHWs receiving small revenues by selling drugs or executing certain tasks can sometimes neglect “unpaid” tasks and be seen as “money driven” by communities [9]. When incentives are not responsive to CHWs’ needs, high turnover can result in a need for continuous efforts to relationship building between CHWs, communities and the health sector. Thus, the socio-economic, cultural and religious contexts shape CHWs’ expectations about incentives. Voluntary CHW programmes could make use of the contextual factors driving volunteerism but at the same time should ensure that incentives are responsive to this context, for example by providing non-financial incentives, such as training, materials such as bicycles, preferential access to healthcare services and recognition via “CHW days” or identity cards [38]. Whether voluntary or paid, CHW programmes require substantial investments. The huge variety in possible incentives—both from the side of the community and health sector—makes the division between paid and voluntary programmes less defined and perhaps less important; incentives should be offered taking into account the interactions between hardware, software and context.

## Conclusions

The above examples show that it is important to investigate how CHW programmes can be shaped to increase common understanding, improve relationships and balance power between different actors in the health system. Questions about “how” and “why” interventions work or do not work are closely related to the software elements. There is a need for mixed method studies that compare the effects of and experiences with different modalities of, for example, supervision and incentives, as well as studies that compare similar modalities in different contexts. This could help to “unpack” enabling and disabling environments related to issues such as trust and power and yield evidence on specific pathways that could positively influence the transactional social processes that shape CHW performance. The conceptual framework presented in this analytical assessment provides guidance on these efforts and should be further tested and refined.

Although there is an increasing body of literature on factors influencing CHW motivation from the perspective of the health sector, there is still a lack of research that gains in-depth insight into the realities of the lives of CHWs and the communities they serve [35, 39, 40]. This resonates with the call regarding the importance of people-centred health systems and thus people-centred science that takes into account that health systems are complex and adaptive systems [41], thriving on mutual trust, dialogue and reciprocity, and their effectiveness correlates with the strengths and nature of the relationships between all health system actors [42]. When the research community complements questioning how things work from the sole viewpoint of the health sector with listening to the voices of CHWs and communities, we would be better able to identify and trigger the mechanisms that can lead to improved CHW performance. Then, the benefit of CHWs’ unique intermediary position between communities and the health sector could be optimized and their role in achieving universal health coverage enhanced.

## Abbreviations

CHW: Community health worker
LMIC: Low- and middle-income country

## Footnotes

1 REACHOUT is an ambitious 5-year international research consortium funded by the European Commission, aiming to generate knowledge to strengthen the performance of CHWs and other close-to-community providers of promotional, preventive and curative primary health services in rural and urban areas in Bangladesh, Ethiopia, Indonesia, Kenya, Malawi and Mozambique.
